# Versorgung älterer Intensivpatienten: Pflegerische Kernaufgaben und zentrale Versorgungsschwerpunkte

**DOI:** 10.1007/s00063-026-01431-8

**Published:** 2026-03-30

**Authors:** Denise Schindele, Carsten Hermes, Tilmann Müller-Wolff

**Affiliations:** 1 Institut für Pflegewissenschaft und -pflegepraxis, Zentrum für Public Health und Versorgungsforschung, Paracelsus Medizinische Privatuniversität, Strubergasse 21, 5020 Salzburg, Österreich; 2 Regionale Kliniken Holding Ludwigsburg, RKH-Akademie, Markgröningen, Deutschland; 3 Brooks College of Health, University of North Florida, Jacksonville, FL USA; 4 Hochschule für Angewandte Wissenschaften Hamburg (HAW Hamburg), Hamburg, Deutschland; 5 Akkon-Hochschule für Humanwissenschaften, Berlin, Deutschland

**Keywords:** Intensivpflege, Gebrechlichkeit, Geriatrie, Intensivstationen, Altersgerecht, Critical care nursing, Frailty, Geriatrics, Intensive care units, Age friendly

## Abstract

**Hintergrund:**

Ältere Menschen (≥ 65 Jahre) werden zunehmend intensivmedizinisch behandelt und weisen häufig geriatrische Syndrome wie Frailty sowie funktionelle Einschränkungen auf. Diese Faktoren erhöhen die Vulnerabilität und beeinflussen die Prognose und den pflegerischen Versorgungsbedarf. Pflegefachpersonen übernehmen dabei zentrale Aufgaben, die wesentlich zum Funktionserhalt und zur Prävention von Komplikationen beitragen.

**Ziel:**

Darstellung zentraler Versorgungsschwerpunkte für ältere Intensivpatienten und Analyse der daraus ableitbaren pflegerischen Kernaufgaben.

**Material und Methode:**

Eine systematische Literaturrecherche in PubMed (U.S. National Library of Medicine) und Cumulative Index to Nursing and Allied Health Literature (CINAHL) identifizierte 11 Studien und Empfehlungen zur Versorgung intensivpflichtiger älterer Menschen. Relevante pflegerische Aufgaben und Versorgungsschwerpunkte wurden thematisch geclustert und narrativ synthetisiert.

**Ergebnisse:**

Pflegerelevante Versorgungsschwerpunkte, die sich in thematische Cluster, wie Delir- und Kognitionsmanagement, Schmerztherapie, Mobilisation, geriatrisches Assessment, Ernährungsmanagement und Risiken im Umgang mit Polypharmazie sowie Schlaf‑/Orientierungsunterstützung, zusammenfassen lassen, konnten identifiziert werden. Pflegefachpersonen übernehmen in diesen Bereichen kontinuierliches Monitoring, alltagsnahe Interventionen sowie die Unterstützung klinischer Entscheidungsprozesse.

**Schlussfolgerung:**

Altersgerechte Versorgungskonzepte auf Intensivstationen („age-friendly intensive care unit [ICU]“) können Funktionserhalt und Delirprävention unterstützen. Die Integration evidenzbasierter Prozesse und klar definierte Abläufe stellen zentrale Bestandteile einer an den Bedürfnissen älterer Intensivpatienten orientierten Versorgung dar.

**Zusatzmaterial online:**

Die Online-Version dieses Beitrags (10.1007/s00063-026-01431-8) enthält umfangreiches Zusatzmaterial.

## Abstract (en)

**Background:**

Older adults represent a growing and highly vulnerable intensive care unit (ICU) population due to geriatric syndromes, functional decline, and multimorbidity. These factors increase the complexity of care and require nursing interventions that support functional stability, prevent complications and address age-related needs.

**Objectives:**

To summarize key care components relevant for older ICU patients and to identify the core nursing responsibilities within these care structures.

**Materials and methods:**

A systematic literature search in PubMed (U.S. National Library of Medicine) and Cumulative Index to Nursing and Allied Health Literature (CINAHL) identified 11 studies and recommendations addressing the care of critically ill adults aged ≥ 65 years. Relevant care components were thematically clustered and the associated nursing responsibilities narratively synthesized.

**Results:**

Several central care priorities were identified, including delirium and cognitive management, pain management, early mobilization, geriatric assessment, nutritional support and medication management, and sleep/comfort support. These components can be grouped into thematic clusters that highlight the central role of nursing in continuous monitoring, nonpharmacological interventions, functional support, and contributing to clinical decision-making.

**Conclusion:**

Age-friendly ICU concepts may support functional preservation and reduce complications such as delirium. Structured, evidence-based processes and clearly defined nursing roles are essential for high-quality, age-appropriate care of older intensive care patients.

Ältere Menschen bilden eine besonders vulnerable Gruppe in der intensivpflegerischen und intensivmedizinischen Versorgung. Geriatrische Syndrome wie Frailty und funktionelle Einschränkungen erhöhen die Komplexität der Versorgung. Dieser Beitrag identifiziert zentrale Versorgungsschwerpunkte und leitet daraus pflegerische Kernaufgaben für eine altersgerechte Intensivversorgung ab.

## Hintergrund

Mit steigender Lebenserwartung nimmt der Anteil älterer Patienten auf Intensivstationen kontinuierlich zu. Geriatrische Syndrome wie Frailty, funktionelle Einschränkungen und Multimorbidität erhöhen die Vulnerabilität, beeinflussen die Prognose und führen zu einem erhöhten Risiko für Komplikationen, wie Delir oder funktionellen Abbau [13, 22, 28, 32, 34]. Die Evidenzlage zur spezifischen Versorgung älterer intensivpflichtiger Menschen bleibt jedoch begrenzt [32, 34].

Es existiert keine einheitliche Definition des „älteren Patienten“, jedoch wird häufig ein Alter ≥ 65 Jahre zugrunde gelegt [24]. Das chronologische Alter allein bildet die Heterogenität älterer Menschen nur begrenzt ab. Funktionsniveau, Frailty und kognitive Fähigkeiten sind für die pflegerische Einschätzung und die Planung des Versorgungsbedarfs wesentlich [3, 24, 27].

## Altersbedingte physiologische Veränderungen

Der altersassoziierte Rückgang funktioneller Reserven betrifft mehrere Organsysteme und gewinnt im kritischen Krankheitsverlauf klinische Relevanz [13].

Wesentliche Veränderungen betreffen:

- kardiovaskuläre Systeme: reduzierte myokardiale Compliance, erhöhter Gefäßwiderstand [17];
- respiratorische Systeme: reduzierte elastische Retraktion und mukoziliäre Clearance [20];
- renale Funktion: Abnahme der glomerulären Filtrationsrate [23];
- Immunfunktion: Immunseneszenz mit inflammatorischen Veränderungen („inflammaging“) [11].

Diese Veränderungen begünstigen geriatrische Syndrome wie Frailty, Sarkopenie und kognitive Einschränkungen, die den intensivmedizinischen Verlauf und unnötige Wiederaufnahmen auf die Intensivstation (ITS) erheblich beeinflussen [12, 13, 18].

## Der ältere Intensivpatient

Ältere intensivpflichtige Menschen weisen gehäuft Frailty, Multimorbidität, Polypharmazie und kognitive Einschränkungen auf [7, 28]. Die zuverlässige Erfassung dieser Faktoren ist im akuten Setting jedoch anspruchsvoll und führt häufig zu unterschätzter Vulnerabilität und verzögerten oder fehladaptierten Entscheidungen [7, 28]. Intensivstationsbezogene Faktoren, wie Immobilität, Schlafstörungen, reduzierte Orientierungsmöglichkeiten und sensorische Überlastung, können Komplikationen, wie Delir oder funktionellen Abbau, begünstigen [29]. Ältere Intensivpatienten berichten häufig über Schmerzen, Angst oder Orientierungslosigkeit, was die Bedeutung strukturierter Kommunikation und spezieller Versorgungsstrukturen unterstreicht [5, 29]. Patienten > 65 Jahren weisen eine erhöhte Mortalität und ein höheres Risiko für nosokomiale Komplikationen auf [6, 13, 19, 26, 31].

Ältere Intensivpatienten berichten häufig über Schmerzen, Angst oder Orientierungslosigkeit

Einige ältere Patienten entwickeln nach einem Intensivaufenthalt anhaltende körperliche, kognitive und psychische Einschränkungen, die das Rehabilitationspotenzial begrenzen [30]. Jedoch zeigt sich ebenfalls, dass funktionelle Stabilität oder sogar Verbesserung möglich sind, sofern rehabilitative, multimodale Strategien frühzeitig implementiert werden [15]. Die Kombination aus erhöhtem Risiko für Komplikationen, funktionellem Abbau und gleichzeitig vorhandenen Rehabilitationsmöglichkeiten macht die Implementierung altersgerechter Versorgungskonzepte zwingend erforderlich [2, 13]. Vor diesem Hintergrund stellt sich die Frage, welche Versorgungsschwerpunkte für ältere Intensivpatienten in der Literatur beschrieben werden und welche pflegerischen Kernaufgaben daraus resultieren.

## Methode

Der vorliegende Beitrag wurde als narrative Übersichtsarbeit mit strukturierter Literaturrecherche konzipiert. Ziel war es, zentrale Versorgungsschwerpunkte sowie pflegerische Kernaufgaben in der intensivmedizinischen Betreuung älterer Menschen (≥ 65 Jahre) zusammenzuführen und einzuordnen. Zwischen Juni und November 2025 erfolgte eine strukturierte Recherche in den Datenbanken PubMed (U.S. National Library of Medicine) und Cumulative Index to Nursing and Allied Health Literature (CINAHL).

Für die Entwicklung der Suchstrings wurden Medical-Subject-Headings(MeSH)-Terms sowie CINAHL Headings verwendet. Der Suchstring beinhaltet die folgenden Schlagwörter: „*aged, elderly, nursing care, critical care nursing, nursing care concepts, intensive care units, care outcomes, critical*“*.* Die Schlüsselbegriffe wurden mittels Boolean-Operatoren kombiniert. Die Suchstrategie wurde iterativ angepasst, um relevante Konzepte zu erfassen.

Die Einschlusskriterien lauteten:

- Patienten ≥ 65 Jahre;
- deutsch- oder englischsprachige Publikationen;
- alle Studiendesigns (quantitativ, qualitativ, Reviews);
- durch Peerreview geprüfte Veröffentlichungen;
- nationale und internationale Empfehlungen/Leitlinien;
- Intensivstation.

Die Ausschlusskriterien lauteten:

- Patienten < 65 Jahre;
- graue Literatur;
- fehlender Peerreview;
- keine Versorgung auf der ITS.

Die Suche wurde durch eine Screeningphase in 2 Stufen ergänzt: Titel- und Abstractscreening; Volltextsichtung anhand der bereits genannten Kriterien.

Relevante Versorgungsbausteine wurden extrahiert, thematisch geclustert und im Sinne einer narrativen Synthese zusammengeführt. Dabei lag der Fokus auf der Ableitung pflegerischer Kernaufgaben innerhalb interprofessioneller Versorgungsstrukturen. Das Suchprotokoll ist als Onlinesupplement A bereitgestellt.

## Versorgung älterer Intensivpatienten

**Abb. 1:**
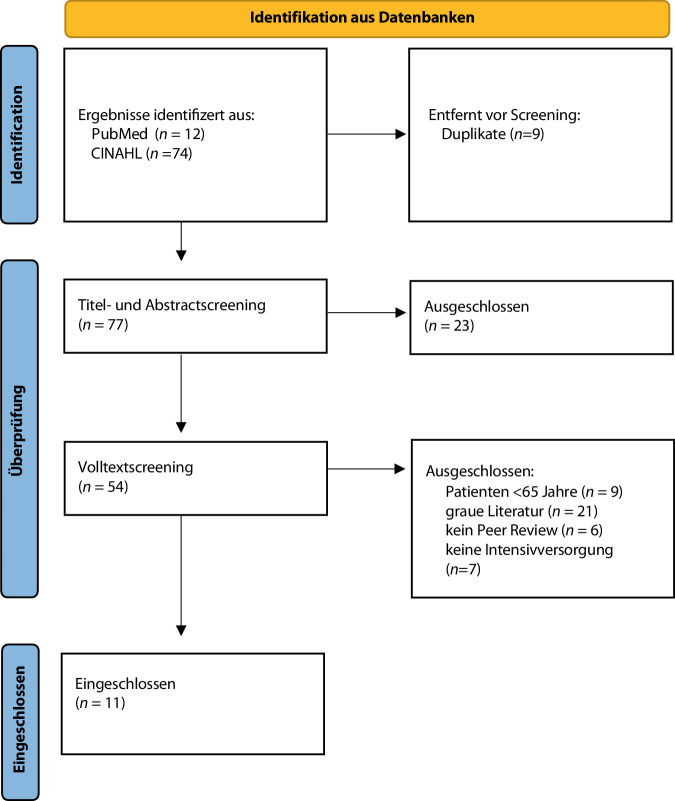
Darstellung der Literaturauswahl in einem PRISMA-Flussdiagramm. *PRISMA* „preferred reporting items for systematic reviews and meta-analyses“. (Modifiziert nach Page et al. [25])

Von 86 Treffern erfüllten 11 Publikationen die Kriterien (Abb. 1). Die Literatur beschreibt Versorgungsschwerpunkte, die sich in thematische Cluster gliedern lassen. Pflegefachpersonen übernehmen im Rahmen interprofessioneller Strukturen die kontinuierliche Beobachtung, Koordination und Umsetzung pflegerelevanter Maßnahmen [3, 29].

Eine altersgerechte Intensivversorgung berücksichtigt 3 Kernelemente [7, 32]:

1. Vulnerabilität,
2. funktionelle Ressourcen,
3. individuelle Ziele und Präferenzen.

**Abb. 2:**
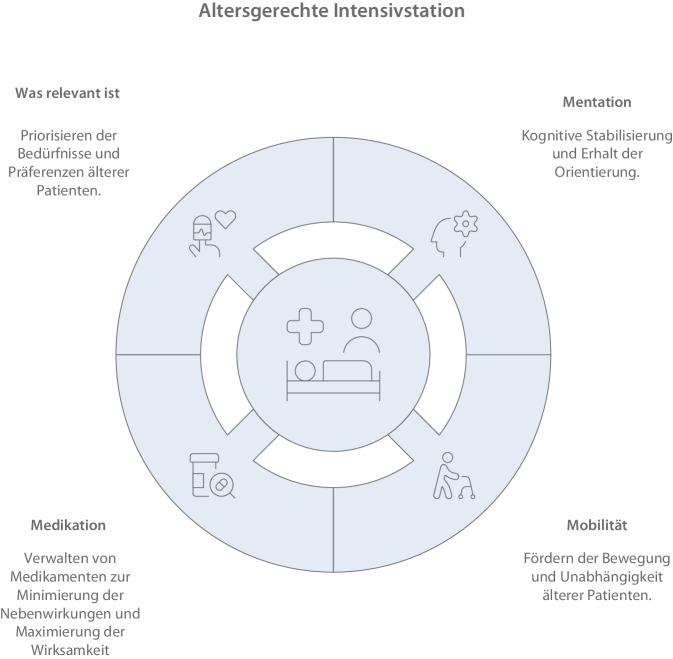
Strukturmodell einer altergerechten Intensivstation. (Erstellt mithilfe künstlicher Intelligenz [Napkin AI, Los Altos, CA, USA])

Multimodale Versorgungskonzepte, die Delirprävention, Schlaf, Mobilität, Ernährung, Schmerztherapie und Orientierung verbinden, gelten als zentral [7, 29, 30]. Die Bedarfe nach Transparenz, emotionaler Unterstützung und Angehörigenintegration verstärken die Bedeutung strukturierter Kommunikation [5, 7, 34]. Die zugrunde liegenden Prinzipien lassen sich konzeptionell 4 zentralen Versorgungsbereichen zuordnen, die eine altersgerechte intensivmedizinische Versorgung strukturieren und entsprechen den Modellen einer altersgerechten Intensivstation (engl. age-friendly-ICU) (Infobox 1; [7, 29, 30]; Abb. 2):

1. Mentation (kognitive Stabilität, Delirprävention)
2. Mobilität (Funktionserhalt, Frühmobilisation)
3. Medikation (Anpassung an geriatrische Vulnerabilität)
4. Was relevant ist („what matters“; Zielklärung, Präferenzorientierung, Angehörigenintegration)

Diese Struktur entspricht in ihrer Logik dem 4M-Prinzip der internationalen Initiative der Age-Friendly Health Systems [16, 21], wird hier jedoch auf den intensivmedizinischen Kontext adaptiert.

### Infobox 1 Konzept der altersgerechten ITS (age-friendly-ICU)

Das Konzept der altersgerechten ITS entstammt der internationalen Initiative der Age-Friendly Health Systems mit dem 4M-Modell („what matters, medication, mentation, mobility“; [16, 21]). Ziel ist es, die besonderen Bedürfnisse älterer Patienten systematisch in Strukturen und Prozesse zu integrieren. Pflegefachpersonen übernehmen hierbei zentrale Rollen in der täglichen Umsetzung [29].

### Versorgungsschwerpunkte

Die Versorgung älterer Intensivpatienten stützt sich auf spezifisch ausgerichtete therapeutische und pflegerische Maßnahmen, deren zentrale Elemente in den folgenden Schwerpunkten zusammengefasst sind und die die Grundlage einer altersgerechten ITS bilden [7, 29].

#### Delir- und Kognitionsmanagement

**Abb. 3:**
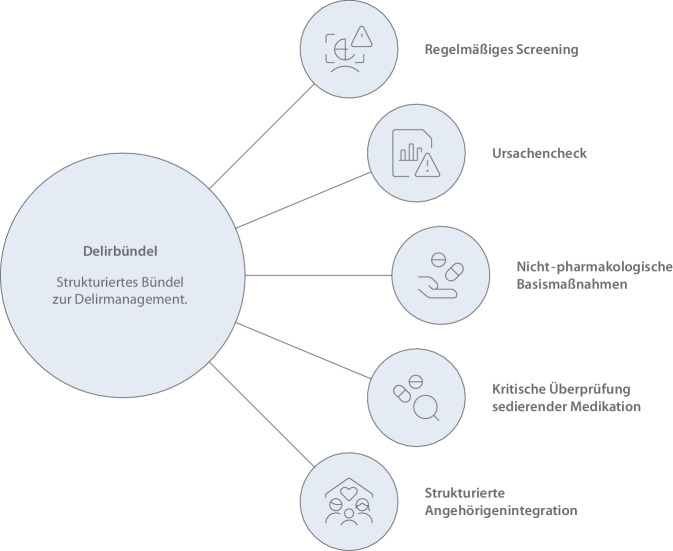
Delirbündel zur Delirprävention. (Erstellt mithilfe künstlicher Intelligenz [Napkin AI, Los Altos, CA, USA])

Delir ist eine der häufigsten Komplikationen älterer Intensivpatienten. Zentrale Elemente der Delirprävention (Abb. 3) sind:

- regelmäßige Delirscreenings [1, 4];
- leitlinienkonforme, individualisierte Sedierungsstrategien mit Ziel einer leichten Sedierung (Richmond Agitation-Sedation Scale [RASS] 0 bis −2) und regelmäßiger Reevaluation [3, 4, 7];
- nichtpharmakologische Maßnahmen, wie Reorientierung, sensorische Unterstützung, Tag-Nacht-Strukturen, vermeiden von Fixierungen und frühe Mobilisation [1, 7, 30].

Ergänzend sollte bei positivem Screening ein strukturierter Ursachencheck (z. B. Infektion, Schmerzen) erfolgen. Sensorische Defizite (Hör‑/Sehbeeinträchtigungen) erhöhen das Delirrisiko; funktionsfähige Hilfsmittel und unterstützende Kommunikationsstrategien sind daher konsequent einzusetzen [3].

Schlafhygienische Maßnahmen (Lärmreduktion, Lichtadaptation, Bündelung pflegerischer Tätigkeiten) tragen nachweislich zur Delirprävention bei und entsprechen häufig geäußerten Bedürfnissen älterer Patienten nach Ruhe und Orientierung [5, 29, 30].

Ältere Patienten haben häufig das Bedürfnis nach Ruhe und Orientierung

Bei vorbestehender Demenz oder chronischer kognitiver Einschränkung ist eine differenzierte Beurteilung erforderlich. Die Einschätzung sollte sich am prämorbiden Status, an Angehörigenangaben und am klinischen Verlauf orientieren, um ein Delir von Baselineveränderungen abzugrenzen [7, 34]. Bei eingeschränkter Kommunikation (Hör‑/Sehdefizite, Aphasie) sind unterstützende Kommunikationsstrategien und Hilfsmittel konsequent einzusetzen [1, 3, 8]. Freiheitsentziehende Maßnahmen sollten nur bei klarer Gefährdung und fehlenden Alternativen erfolgen. Nichtpharmakologische Alternativen (z. B. engmaschige Beobachtung, Anpassung der Umgebung, Einbindung von Angehörigen) sind vorrangig zu prüfen. Indikation, Dauer und Reevaluation sind nachvollziehbar zu dokumentieren [3].

#### Schmerzmanagement

Schmerzen werden bei älteren Patienten häufig unterdiagnostiziert, insbesondere bei kognitiver Einschränkung. Empfohlen wird ein altersadaptierter, multimodaler Ansatz mit dem Grundsatz „start low, go slow“. Pflegefachpersonen übernehmen die Schmerzerfassung, Umsetzung pharmakologischer und nichtpharmakologischer Maßnahmen sowie die Evaluation der Wirksamkeit [3, 9, 29].

Bei kognitiv eingeschränkten oder nichtkommunikationsfähigen Patienten sollten validierte Beobachtungsskalen, wie Beurteilung von Schmerz bei Demenz (BESD) oder Behavioural Pain Scale (BPS), eingesetzt werden [8, 9]. Eine adäquate Analgesie erfordert die Abwägung zwischen dem Risiko von Delir und Atemdepression und den Folgen unzureichend behandelter Schmerzen, die selbst zur Delirentstehung beitragen können [7, 8]. Nichtpharmakologische Maßnahmen, wie Lagerungsoptimierung, Wärme‑/Kälteanwendungen, Reorientierung und ruhige Umgebungsgestaltung, sollten regelhaft ergänzt werden [29].

#### Frühmobilisation und Funktionserhalt

Bei älteren Intensivpatienten ist eine frühe, sichere Mobilisation entscheidend, um Immobilitätsfolgen, wie Muskelabbau, Delir und funktioneller Verlust, vorzubeugen. Protokollgeleitete, interprofessionell abgestimmte Aktivierungsmaßnahmen, einschließlich Physiotherapie und Ergotherapie, sind frühzeitig einzuleiten [3, 7, 29, 33]. Vor Mobilisationsbeginn sollte ein strukturierter Sicherheitscheck erfolgen, der die hämodynamische Stabilität, respiratorische Situation sowie das Fehlen akuter Kontraindikationen berücksichtigt [33].

Ein stufenweises Mobilisationskonzept erleichtert die Zieldefinition und Verlaufskontrolle

Ein stationsspezifisches, stufenweises Mobilisationskonzept (z. B. Bettkante, Stand, Transfer, Gang) erleichtert die Zieldefinition und Verlaufskontrolle. Ergänzend trägt ein interprofessionelles Training nach einem standardisierten Curriculum [10] zur gemeinsamen Zielklärung, sicheren Umsetzung und nachhaltigen Etablierung der Mobilisationsmaßnahmen bei. Zur standardisierten Dokumentation kann die ICU Mobility Scale herangezogen werden [29, 33].

Ergänzend sollten durch die Pflegefachpersonen alltagsnahe Aktivitäten, wie Lagerungswechsel, Transfertraining und der Einsatz individueller Hilfsmittel, gezielt gefördert werden, um die Autonomie und das Rehabilitationspotenzial zu erhalten [7].

#### Geriatrisches Assessment

Eine strukturierte geriatrische Basisbeurteilung bildet die Grundlage einer altersgerechten Intensivversorgung [7]. Die Einschätzung von Frailty, Funktion, Kognition, Ernährung und Medikationsrisiken bildet die Grundlage individueller Therapieentscheidungen [3, 7]. Pflegefachpersonen übernehmen Screening, Datenerhebung, kontinuierliche Beobachtung und die Einbindung ihrer Beobachtungen in die interprofessionelle Entscheidungsfindung [3, 29].

Erhebung des Frailty Status ist als Grundlage für eine individualisierte Zielplanung essentiell

Ein Frailty- und Funktionsscreening sollte möglichst innerhalb der ersten 24 h nach Aufnahme erfolgen. Als praktikables Instrument wird die Clinical Frailty Scale (CFS) empfohlen, die auch im intensivmedizinischen Kontext eingesetzt und validiert wurde [7, 14]. Die Erhebung des prämorbiden Funktionsniveaus sollte strukturiert über Pflegeanamnese, Angaben von Angehörigen sowie verfügbare Vorbefunde erfolgen [29]. Die Einstufung von Frailty und Baselinefunktion beeinflusst die Versorgungsplanung unmittelbar: Mobilisationsziele sind realistisch anzupassen, Delirprävention und zurückhaltende Sedierungsstrategien zu priorisieren sowie Therapieziele frühzeitig interprofessionell zu reflektieren [1, 7]. Frailty ist dabei nicht als Ausschlusskriterium, sondern als Grundlage einer individualisierten Zielplanung zu verstehen.

#### Ernährungsmanagement

Unterernährung, Sarkopenie und Malnutrition sind häufig und prognostisch relevant [1, 7]. Frühzeitige ernährungsmedizinische Expertise, priorisierte enterale Ernährung, engmaschige Bilanzierung und Evaluierung der Verträglichkeit gehören zu den zentralen pflegerischen Aufgaben [1, 7, 29].

Die Ernährung sollte frühzeitig mit funktionellen Zielen verknüpft werden

Im Sinne einer „rehab nutrition“ sollte die Ernährung frühzeitig mit funktionellen Zielen verknüpft werden. Eine bedarfsadaptierte Energie- und Proteinzufuhr sowie die Berücksichtigung eines möglichen Refeeding-Risikos sind insbesondere bei vorbestehender Mangelernährung relevant [1, 12]. Der Zusammenhang zwischen adäquater Nährstoffversorgung, Erhalt von Muskelmasse und Mobilisationsfähigkeit unterstreicht die Bedeutung einer engen Verzahnung von Ernährungsmanagement und aktivierender Pflege [15, 29].

#### Medikamentenmanagement

Polypharmazie erhöht das Risiko für Komplikationen, Stürze und Delir. Eine strukturierte Arzneimittelüberprüfung, bevorzugt in interprofessioneller Zusammenarbeit mit klinischer Pharmazie sowie Orientierung an den Beers-Kriterien (Infobox 2) wird empfohlen. Pflegefachpersonen tragen wesentlich zum Monitoring und zur Früherkennung von Nebenwirkungen bei [3, 4, 7].

Ergänzend können für die europäische Versorgungspraxis auch die Kriterien gemäß Screening Tool of Older Persons’ Prescriptions (STOPP)/Screening Tool to Alert to Right Treatment (START) sowie die Fit-for-the-aged (FORTA)-Liste zur Bewertung potenziell inadäquater Medikation herangezogen werden [1, 3]. Im intensivmedizinischen Kontext ist insbesondere auf sedierende und anticholinerg wirksame Substanzen sowie auf organfunktionsabhängige Dosisanpassungen zu achten, da altersassoziierte pharmakokinetische Veränderungen das Risiko medikationsbedingter Komplikationen erhöhen [7, 13]. Die Medikation sollte in regelmäßigen interprofessionellen Visiten evaluiert werden.

##### Infobox 2 Beers-Kriterien

Die Beers-Kriterien (vollständig: American Geriatrics Society [AGS] Beers Criteria for Potentially Inappropriate Medication Use in Older Adults) stellen eine evidenzbasierte Liste von Arzneimitteln, die bei älteren Menschen ein erhöhtes Risiko für unerwünschte Wirkungen aufweisen und daher vermieden oder mit besonderer Vorsicht eingesetzt werden sollten, dar. Die Kriterien wurden erstmals im Jahr 1991 von Mark H. Beers entwickelt und werden seither regelmäßig durch die AGS aktualisiert (zuletzt im Jahr 2023). Sie dienen als international anerkanntes Instrument zur Beurteilung der Arzneimittelangemessenheit im höheren Lebensalter.

#### Angehörigenintegration

Angehörige unterstützen Orientierung, emotionale Stabilität und Entscheidungsprozesse. Strukturierte Kommunikation und Einbindung verbessern Patientenerleben und reduzieren Angst sowie Orientierungslosigkeit [1, 3, 5]. Pflegefachpersonen unterstützen die kontinuierliche Informationsweitergabe, strukturierte Einbindung von Bezugspersonen in Versorgungsmaßnahmen sowie die Teilnahme an Gesprächsprozessen [29].

#### Management invasiver Zugänge

Ältere Patienten haben ein erhöhtes Risiko für infektions- und mobilitätsassoziierte Komplikationen. Empfohlen werden klare Indikationsstellung, tägliche Überprüfung der Notwendigkeit und frühzeitige Entfernung invasiver Zugänge. Eine tägliche Evaluation im interprofessionellen Team sollte Teil der täglichen Visite zwischen Intensivmedizinern und Pflegefachpersonen sein [1].

#### Transition und funktionelle Zielorientierung

Neben der Vermeidung akuter Komplikationen sollte das Behandlungsziel älterer Intensivpatienten auf den Erhalt bzw. die Wiederherstellung funktioneller Selbstständigkeit ausgerichtet sein [7, 15]. Funktionelle Zielparameter, wie Mobilitätsstatus, Selbstständigkeit in Aktivitäten des täglichen Lebens (ADL) und kognitive Stabilität, sind insbesondere im Übergang von der Intensivstation in weiterführende Versorgungsstrukturen relevant [32]. Ein strukturiertes Entlassmanagement unterstützt die Kontinuität der Versorgung beim Transfer von der Intensivstation auf die Normalstation sowie in Rehabilitations- oder Pflegeeinrichtungen. Pflegefachpersonen übernehmen hierbei die Bewertung des aktuellen Funktionsstatus, die strukturierte Informationsweitergabe sowie die Einbindung von Angehörigen in weitere Versorgungsprozesse [3, 29]. Zur Sicherung funktioneller Stabilität sollten Mobilitätsstatus, Delirstatus, aktuelle Medikation und Ernährungsplanung vor Verlegung klar dokumentiert und kommuniziert werden [7, 29]. Eine beispielhafte Checkliste zur Entlassung liegt diesem Artikel im Onlinesupplement B bei.

#### Strukturierte Umsetzung im klinischen Alltag

Die Umsetzung altersgerechter Intensivversorgung erfordert klar definierte, schichtbezogene Abläufe. Aus der Literatur lassen sich 5 zentrale Prozessdimensionen ableiten: Mentation, Mobilität, Schmerz/Sedierung, Schlaf/Orientierung und Medikation [7, 29, 30].

Multimodale Delirprävention, strukturierte Schlafprotokolle, frühe Mobilisation sowie angepasste Analgosedierung werden als zentrale Elemente einer altersgerechten Intensivversorgung beschrieben [29, 30]. Ebenso wird die Notwendigkeit einer systematischen geriatrischen Risikostratifikation, einschließlich Frailty-Assessment, funktioneller Evaluation und strukturierter Medikationsanalyse als zentrale Elemente der Versorgung von älteren Intensivpatienten, hervorgehoben [1].

Diese Konzepte entsprechen in ihrer Struktur dem Ansatz einer altersgerechten ITS, indem sie geriatrische Kernprinzipien systematisch in den intensivmedizinischen Kontext übertragen [29, 30].

Zur praktischen Anwendung ist in Tab. 1 eine strukturierte Assessmenttoolbox dargestellt.

**Tab. 1:** Assessmenttoolbox in der altersgerechten Intensivversorgung

Versorgungsbereich	Empfohlenes Assessmentinstrument	Zeitpunkt/Häufigkeit	Verantwortlichkeit	Ziel	Quelle
*Frailty*	CFS	Bei Aufnahme auf die ITS	Pflegefachpersonen (Erhebung) + ärztliche Validierung	Risikostratifizierung, Prognoseabschätzung	[1, 3, 7, 14, 29]
*Mentation/Delir*	CAM-ICU oder ICDSC	Mindestens einmal pro Schicht und bei Veränderung Bewusstsein	Pflegefachpersonen	Früherkennung, Delirprävention	[1, 3, 4, 7, 8]
*Sedierung*	RASS	Mindestens einmal pro Schicht und bei Veränderung Bewusstsein	Pflegefachpersonen	Vermeidung von Übersedierung	[1, 3, 8, 30]
*Schmerz*	NRS, BESD, BPS	Mindestens einmal pro Schicht und vor und nach Prozeduren/Interventionen	Pflegefachpersonen	Adäquate Analgesie	[1, 3, 8, 9, 30]
*Mobilität*	IMS	Täglich	Pflegefachpersonen, Physiotherapie, interprofessionelles Team	Funktionserhalt, Dekonditionierungsprophylaxe	[1, 3, 7, 30, 33]
*Ernährung*	Nutritional Risk Screening	Bei Aufnahme + im Verlauf	Pflegefachpesonen, Ernährungsberatung	Prävention von Mangelernährung	[1, 7, 29]
*Medikation/Polypharmazie*	Strukturierte Medikationsanalyse (Beers-Kriterien, STOPP/START, FORTA)	Bei Aufnahme + im Verlauf	Ärztlich, pflegerische Beobachtung von Nebenwirkungen	Reduktion anticholinerger/sedierender Last	[1, 3, 4, 7]

*BESD* Beurteilung von Schmerz bei Demenz, *BPS* Behavioural Pain Scale, *CAM-ICU* Confusion Assessment Method for the Intensive Care Unit, *CFS* Clinical Frailty Scale, *ICDSC* Intensive Care Delirium Checklist, *FORTA *„fit for the aged“, *ITS* Intensivstation, *NRS* numerische Rating-Skala, *IMS* ICU Mobility Scale, *RASS* Richmond Agitation-Sedation Scale, *STOPP* Screening Tool of Older Persons’ Prescriptions, *START* Screening Tool to Alert to Right Treatment

## Diskussion

Die vorliegende Übersichtsarbeit verdeutlicht, dass ältere intensivpflichtige Menschen eine vulnerable und zugleich heterogene Patientengruppe darstellen, deren Versorgungsbedarf wesentlich von geriatrischen Syndromen, funktionellen Einschränkungen und Multimorbidität beeinflusst wird [7, 32]. Die identifizierten Versorgungsschwerpunkte verdeutlichen, dass eine systematische Berücksichtigung von funktionellen Ressourcen und Vulnerabilität für ein verbessertes Behandlungsergebnis wesentlich ist [3, 29]. Die eingeschlossene Literatur hebt besonders die Bedeutung nichtpharmakologischer, pflegezentrierter Maßnahmen, wie Delir- und Kognitionsmanagement, Schmerztherapie und Frühmobilisation, hervor [7, 29, 30]. Diese Schwerpunktbereiche entsprechen zugleich den Kerninhalten von Konzepten einer altersgerechten ITS, die eine strukturierte, auf ältere Patienten abgestimmte Versorgung anstreben und pflegerische Interventionen als zentrale Bestandteile definieren [29]. Gleichzeitig weist die Literatur auf relevante Umsetzungsbarrieren hin, darunter eingeschränkte personelle Ressourcen, fehlende Standardisierung und unzureichende Schulungsstrukturen, die die Umsetzung evidenzbasierter Maßnahmen im klinischen Alltag erschweren [3, 29]. Die Ergebnisse unterstreichen zudem die Schlüsselrolle der Pflegefachpersonen, die durch kontinuierliche Beobachtung, Durchführung und Evaluation pflegerischer Interventionen maßgeblich zur Umsetzung altersfreundlicher Versorgungskonzepte beitragen [3, 29].

Obwohl die Wirksamkeit einiger Maßnahmen gut beschrieben ist, zeigt die Literatur deutliche Heterogenität. Ein Großteil der eingeschlossenen Ergebnisse basiert auf Beobachtungsdesigns oder Expert Consensus Statements, wodurch kausale Zusammenhänge nur eingeschränkt ableitbar sind [3, 7]. Einige Interventionen werden zudem häufig als Bestandteil multimodaler ITS-Bündel untersucht, sodass isolierte Effekte einzelner Maßnahmen schwer zu bestimmen sind [29, 30]. Auch die Operationalisierung zentraler Konzepte, wie Frailty oder funktioneller Status, variiert zwischen Studien, was die Vergleichbarkeit der Ergebnisse einschränkt [7].

Die Implementierung standardisierter, pflegegetragener Prozesse rückt in den Fokus

Damit rückt die Implementierung standardisierter, pflegegetragener Prozesse in den Fokus. Die Umsetzung strukturierter Prozesse kann wesentlich dazu beitragen, altersbedingte Risiken zu minimieren und funktionelle Ressourcen zu erhalten [3, 29]. Das Konzept der altersgerechten ITS bietet hierfür einen geeigneten Rahmen, da es pflegerische Kernelemente in ein strukturiertes Versorgungsmodell integriert und Veränderungen auf Struktur- und Prozessebene anstoßen kann [29].

Für die Forschung besteht erheblicher Bedarf an Studien, die pflegerische Interventionen gezielt untersuchen. Relevante Fragestellungen betreffen die isolierte Wirksamkeit einzelner Maßnahmen, wie sensorische Unterstützung oder Schlafhygiene, die Validität und Anwendbarkeit standardisierter Frailty- und Funktionsscreenings auf der Intensivstation, den Einfluss einer frühzeitigen pflegeinitiierten Mobilisation auf funktionelle Outcomes sowie die Rolle von patientenzentrierter Kommunikation und Angehörigenintegration. Darüber hinaus sollten Implementierungs- und Wirksamkeitsstudien zu Modellen einer altersgerechten ITS durchgeführt werden. Randomisierte oder quasi-experimentelle Designs wären notwendig, um robustere Evidenzgrundlagen für pflegerische Kerninterventionen zu schaffen.

## Limitationen

Die heterogene Evidenzlage und die begrenzte Zahl spezifischer Studien zu pflegerischen Interventionen stellen Limitationen dar. Eine formale Qualitätsbewertung wurde nicht durchgeführt; die Ergebnisse sind als konzeptionelle Orientierung zu verstehen.

## Fazit für die Praxis

- Die Vulnerabilität der älteren Patienten sollte systematisch erfasst und in die Entscheidungsfindung miteinbezogen werden.
- Maßnahmen, wie Delir- und Schmerzmanagement, Mobilisation und der Erhalt funktioneller Ressourcen, sollten in der Versorgung priorisiert werden.
- Angehörigenintegration und klare Kommunikation sollte gefördert werden.

## Supplementary Information

ESM 1_online Supplement A: Rechercheprotokoll

ESM 2_online Supplement B: Entlasscheckliste
