# Supplementary material for: Intensive care of elderly patients: Core nursing responsibilities and central care priorities
Source: Med Klin Intensivmed Notfmed. 2026 Mar 30;121(4):281–9. [Article in German] doi: 10.1007/s00063-026-01431-8 (PMC13133203; doi:10.1007/s00063-026-01431-8)
Supplement: Supplementary file 1 — ESM 1_online Supplement A: Rechercheprotokoll [file 63_2026_1431_MOESM1_ESM.pdf]

Supplement A

Rechercheprotokoll für eine systematische Literaturrecherche

Generelle Informationen zur Entwicklung der Suchstrategie 2
Forschungsfrage(n) 2
Ein- und Ausschlusskriterien 2
1 Festlegung des Rechercheprinzips 2
2 Festlegung der Suchkomponenten 2
3 Festlegung der zu durchsuchenden Datenbanken 3
4 Identifikation von Stichwörtern 3
5 Identifikation von Schlagwörtern 4
6 Entwicklung des Suchstrings 5
8 Durchführung der Recherche 6
9 Dokumentation der Recherche 7
9.1 Pubmed 7
9.2 CINAHL 7

Nutzungsinformationen:

Das Rechercheprotokoll ist eine Beilage zu RefHunter. Es dient der systematischen und transparenten Entwicklung und Dokumentation einer Literaturrecherche. Der Aufbau orientiert sich an der Vorgehensweise, die in den zehn Rechercheschritten von RefHunter beschrieben ist. Das Rechercheprotokoll steht zur kostenlosen Nutzung zur Verfügung (www.refhunter.org).

Zitiervorschlag:

Hirt, J., Nordhausen, T. (2022). Rechercheprotokoll für eine systematische Literaturrecherche. In: Nordhausen, T., Hirt, J. RefHunter. Systematische Literaturrecherche. https://refhunter.org/research_support/rechercheprotokoll/ [Zugriff am: Datum]

Version 4.0
30. Juni 2022

BY SA

Generelle Informationen zur Entwicklung der Suchstrategie

Name(n) der die Suchstrategie entwickelnden Person(en): Denise Schindele
Datum oder Zeitraum der Entwicklung: Juni 2025 – November 2025

Forschungsfrage(n)

Notierung der Forschungsfrage(n): Welche pflegerischen Versorgungskonzepte haben Einfluss auf das Outcome von älteren Patienten (>65 Jahre) auf der Intensivstation?

Which nursing care concepts improve the outcome of patients aged ≥65 years in the intensive care unit?

Ein- und Ausschlusskriterien

Domäne	Einschlusskriterien	Ausschlusskriterien
**Domäne 1: keine Einschränkung**	Systematic, Metaanalysen, Observational, RCT, clinical Trials, qualitatvie Studien, Case Reports, Leitlinien, Empfehlungen	Kongressbereichte, graue Literatur
**Domäne 2: erwachsene, ältere Patienten**	Patienten ≥65 Jahre	Patienten <65 Jahre
**Domäne 3: pflegerische Versorgungskonzepte**	Pflegerische Versorgungskonzepte	Keine Pflege benannt
**Domäne 4: Outcome**	Mortalität, Länge des ICU Aufenthaltes, Komplikationen jeglicher Art auf der ICU	
**Domäne 5: Intensivstation**	Intensivstation	Keine Intensivstation

Begründung für (einzelne) Ein- und Ausschlusskriterien:

☐ Begründung anführen.

☒ Keine Begründung notwendig.

1 Festlegung des Rechercheprinzips

Begründung für (einzelne) Ein- und Ausschlusskriterien:

☐ Sensitives Rechercheprinzip
Ziel: Möglichst alle relevanten Treffer finden.

☐ Spezifisches Rechercheprinzip
Ziel: Möglichst schnell die wichtigsten relevanten Treffer finden.

☒ Mischform (teilweise sensitiv bzw. spezifisch)
Ziel: Möglichst viele relevante Treffer mit einem optimierten Aufwandsverhältnis finden.

2 Festlegung der Suchkomponenten

Suchkomponente	Bezeichnung
**Suchkomponente 1**	Patienten >65 Jahre
**Suchkomponente 2**	Pflegerische Versorgungskonzepte
**Suchkomponente 3**	Outcome
**Suchkomponente 4**	Intensivstation

Begründung für (einzelne) festgelegte oder nicht festgelegte Suchkomponenten:

☐ Begründung anführen.

☒ Keine Begründung notwendig.

3 Festlegung der zu durchsuchenden Datenbanken

Datenbank	Bezeichnung<br>Begründung
Datenbank 1	Pubmed
Datenbank 2	CINHAL

4 Identifikation von Stichwörtern

Suchkomponenten	Stichwörter
**Suchkomponente 1: Patienten >65**	ELDERLY<br>ELDERLY PATIENT<br>PATIENT AGED >65 YEARS
**Suchkomponente 2: pflegerische Versorgungskonzepte**	NURSING CARE<br>CRITICAL CARE NURSING<br>CARE CONCEPTS<br>NURSING TREATMENTS
**Suchkomponente 3: Intensivstation**	INTENSIVE CARE UNIT
**Suchkomponente 4: Outcome**	CARE OUTCOMES, CRITICAL

Begründung für (einzelne) gewählte oder nicht gewählte Stichwörter:

☐ Begründung anführen.

☒ Keine Begründung notwendig.

5 Identifikation von Schlagwörtern

Stichwörter	Suchkomponenten	Schlagwörter: PubMed	Schlagwörter: CINAHL
ELDERLY<br>ELDERLY PATIENT<br>PATIENT AGED >65 YEARS	**Suchkomponente 1: Patienten >65**	AGED<br>ELDERLY	AGED<br>ELDER<br>GERIATRIC<br>OLDER
NURSING CARE<br>CRITICAL CARE<br>NURSING<br>CARE CONCEPTS<br>NURSING TREATMENTS	**Suchkomponente 2: pflegerische Versorgungskonzepte**	NURSING CARE<br>CRITICAL CARE<br>NURSING<br>NURSING<br>CARE CONCEPTS	CRITICAL CARE
INTENSIVE CARE UNIT	**Suchkomponente 3: Intensivstation**	INTENSIVE CARE UNITS	INTENSIVE CARE UNITS
OUTCOME	**Suchkomponente 4: Outcome**	CARE OUTCOMES, CRITICAL	CRITICAL CARE OUTCOMES

Begründung für (einzelne) gewählte oder nicht gewählte Schlagwörter:

☒ Begründung anführen.

☐ Keine Begründung notwendig.

CARE OUTCOME, CRITICAL: Ein Maß für die Mortalitäts- und Morbiditätsraten bei Patienten, die eine KRITISCHE VERSORGUNG und INTENSIVVERSORGUNG erhalten, ermittelt anhand des Datums der Entlassung aus dem Krankenhaus.

6 Entwicklung des Suchstrings

Suchkomponente	Suchstring: **Pubmed**	Suchstring: **CINAHL**
**Suchkomponente 1: Bezeichnung**	AGED 1 OR<br>ELDERLY	AGED<br>ELDER<br>GERIATRIC<br>OLDER
	AND	AND
**Suchkomponente 2: Bezeichnung**	NURSING CARE OR<br>CRITICAL CARE NURSING<br>NURSING OR<br>CARE CONCEPTS	CRITICAL CARE
	AND	AND
**Suchkomponente 3: Bezeichnung**	INTENSIVE CARE UNIT	INTENSIVE CARE UNIT
	AND	AND
**Suchkomponente 4: Outcome**	CARE OUTCOMES, CRITICAL	CRITICAL CARE OUTCOME

Begründung für (einzelne) verwendete oder nicht verwendete Suchtechniken und/oder Stich- und Schlagwörter

☐ Begründung anführen.

☒ Keine Begründung notwendig.

8 Durchführung der Recherche

Dokumentation der Suchfilter (bspw. in Hinblick auf die Ein- und Ausschlusskriterien für die Studienauswahl), die bei der Recherche angewendet werden sowie Besonderheiten, die bei der Durchführung der Recherche in Datenbanken auftraten.

Alle Zeiträume

Aged >65 Jahre

Englisch/Deutsch

Peer Reviewed Artikel

Wissenschaftliche Zeitschrift

9 Dokumentation der Recherche

Dokumentation der datenbankspezifischen Suchstrings mit Angabe des Suchdatums und der Suchtreffer.

9.1 Pubmed

Suchdatum: 28.06.2025 – 09.11.2025

Suchstring online hinterlegt: ☐ Ja
☒ Nein

#	Eingabe	Trefferzahl
**1**	(((((((((AGED[MESH TERMS]) OR (ELDERLY[MESH TERMS])) AND (NURSING CARE[MESH TERMS])) OR (CRITICAL CARE NURSING[MESH TERMS])) OR (CARE CONCEPTS)) OR (NURSING)) AND (ICU INTENSIVE CARE UNITS[MESH TERMS])) AND (CARE OUTCOMES, CRITICAL[MESH TERMS]))	12

9.2 CINAHL

Suchdatum: 28.06.2025 – 09.11.2025

Suchstring online hinterlegt: ☐ Ja
☒ Nein

#	Eingabe	Trefferzahl
**1**	INTENSIVE CARE UNIT AND (ELDERLY OR AGED OR OLDER OR ELDER OR GERIATRIC) AND CRITICAL CARE AND CRITICAL CARE OUTCOMES	74
