# Supplementary material for: Intensive care of elderly patients: Core nursing responsibilities and central care priorities
Source: Med Klin Intensivmed Notfmed. 2026 Mar 30;121(4):281–9. [Article in German] doi: 10.1007/s00063-026-01431-8 (PMC13133203; doi:10.1007/s00063-026-01431-8)
Supplement: Supplementary file 2 — ESM 2_online Supplement B: Entlasscheckliste [file 63_2026_1431_MOESM2_ESM.pdf]

Supplement B: Entlass-Checkliste, 48h vor geplanter Entlassung

Bereich	Prüfpunkte	✓
**Funktion & Mobilität**	Mobilitätsstatus dokumentiert (z. B. IMS)	☐
	Aktuelles Mobilisationsniveau festgelegt (Bettkante/Stand/Transfer/Gang)	☐
	ADL-Status eingeschätzt	☐
	Weiterführende Mobilisationsziele definiert	☐
**Kognition & Delir**	Delirstatus dokumentiert (z. B. CAM-ICU / ICDSC)	☐
	Baseline-Kognition berücksichtigt (Angehörigenangaben)	☐
	Sedierungsniveau stabil (leichte Sedierung oder wach)	☐
	Delirpräventionsmaßnahmen organisiert	☐
**Medikation**	Gesamtmedikation überprüft	☐
	Sedierende / anticholinerge Substanzen kritisch geprüft	☐
	Dosisanpassung bei Organfunktionsstörung kontrolliert	☐
	Nicht mehr indizierte Akutmedikation beendet	☐
**Ernährung**	Ernährungsstatus dokumentiert	☐
	Ernährungsplan (oral/enteral/parenteral) definiert	☐
	Weiterbehandlung abgestimmt	☐
**Hilfsmittel & Pflegebedarf**	Mobilitäts-/Pflegehilfsmittel organisiert	☐
	Sensorische Hilfsmittel verfügbar	☐
	Dekubitus-/Sturzrisiko bewertet	☐
**Angehörige & Transition**	Angehörigenbriefing erfolgt	☐
	Therapieziele dokumentiert	☐
	Schriftliche Übergabe an Zielbereich erfolgt	☐

Eigendarstellung auf der Grundlage [7, 14, 26]
